# TM7SF2 regulates cell proliferation and apoptosis by activation of C-Raf/ERK pathway in cervical cancer

**DOI:** 10.1038/s41420-021-00689-5

**Published:** 2021-10-19

**Authors:** Yichi Xu, Xin Chen, Shuya Pan, Zhi-wei Wang, Xueqiong Zhu

**Affiliations:** Center for Uterine Cancer Diagnosis & Therapy Research of Zhejiang Province, Department of Obstetrics and Gynecology, The Second Affiliated Hospital of Wenzhou Medical University, Wenzhou, People’s Republic of China

**Keywords:** Tumour biomarkers, Cervical cancer

## Abstract

Transmembrane 7 superfamily member 2 (TM7SF2) coding an enzyme involved in cholesterol metabolism has been found to be differentially expressed in kinds of tissues. Nevertheless, the role of TM7SF2 in the regulation of growth and progression among various cancers is unclear. In this study, the immunohistochemistry (IHC) assay, real-time RT-PCR and western blotting analysis were used to determine the TM7SF2 expression in cervical cancer tissues. Next, we used multiple methods to determine the ability of cell proliferation, migration, invasion, apoptosis, and cell cycle in cervical cancer cells after TM7SF2 modulation, such as CCK8 assay, colony formation assay, Transwell assay, wound healing assay, and flow cytometry. Our results revealed that upregulation of TM7SF2 facilitated cell proliferation and metastasis, suppressed cell apoptosis and prevented G0/G1 phase arrests in C33A and SiHa cells. Consistently, the opposite effects were observed after TM7SF2 knockout in cervical cancer cells. Further, we found that TM7SF2 participated in promoting tumorigenesis and progression via activation of C-Raf/ERK pathway in cervical cancer, which can be partly reversed by Raf inhibitor LY3009120. Moreover, TM7SF2 overexpression contributed to enhancement of xenograft tumor growth in vivo. Our findings indicated that TM7SF2 plays a vital role in tumor promotion by involving in C-Raf/ERK activation. Therefore, TM7SF2 could serve as a therapeutic target in future cervical cancer treatment.

## Introduction

Cervical cancer is the fourth leading malignant tumor among women worldwide, and it is estimated that there are more than 600,000 new cervical cancer cases and 340,000 deaths worldwide in 2020 [1]. As a result of extensive cytological screening and the widespread use of HPV vaccine, the incidence and mortality of cervical cancer have decreased significantly [2]. The occurrence of cervical cancer is related to a series of risk factors. The most important risk factor is HPV infection. Persistent HPV infection, especially HPV 16 and 18 types, is the most important cause of cervical cancer development [3]. In addition to HPV infection, the imbalance expression of related genes and pathways is also the basic feature of cervical carcinogenesis [4]. There are several approaches to prevent and treat cervical cancer, such as HPV vaccines, surgical treatments, chemotherapy, and radiotherapy. Nevertheless, advanced cervical cancer often has drug resistance and a strong invasiveness and metastasis, leading to treatment failure. It is estimated that there will be 14,480 new cancer cases and 4290 deaths of cervical cancer in the United States in 2021 [5]. Therefore, exploring the relevant mechanisms of cervical tumorigenesis can provide new ideas for us to discover potential biomarkers for the prediction of diagnosis and prognosis and to develop new targeted drugs for the treatment of cervical cancer.

Transmembrane 7 superfamily member 2 (TM7SF2) is located in chromosome 11q13 and encoded the 3β-hydroxysterol Δ14-reductase, which is an enzyme participated in cholesterol biosynthesis [6, 7]. TM7SF2 mRNA is differently expressed in adult heart, brain, pancreas, lung, liver, skeletal muscle, kidney, ovary, prostate, and testis tissues, but it is not expressed in placenta, spleen, thymus, small intestine, colon, or peripheral blood leukocytes [6]. The deficiency of TM7SF2 leads to the suppression of NF-κB signaling pathway and impairs inflammatory response in renal [8]. Notably, a previous study had revealed that the expression of TM7SF2 was different between the non-aggressive follicular carcinomas and the aggressive follicular carcinomas by gene expression profiling, indicating that TM7SF2 may be used to differentiate between non-aggressive and aggressive follicular carcinomas [9]. Additionally, it has been showed a delayed cell cycle of liver cell progression at the G1/S phase during liver regeneration in TM7SF2 knockout mice, contributing to the decrease of cell division. Furthermore, the expression of tumor suppressor p53 is constantly and drastically increased after partial hepatectomy in TM7SF2 knockout mice [10].

Nevertheless, the role of TM7SF2 is unclear in multiple kinds of cancers and further investigate is needed. Extracellular signal–regulated kinases (ERK) pathway, one of the mitogen-activated protein kinases (MAPK) pathways, is dysregulated in many human malignancies [11]. The activation of ERK1/2 pathway modulated cell proliferation, migration, and angiogenesis by its phosphorylation [11]. Raf kinases (Raf) are composed of three subtypes, A-Raf, B-Raf, and C-Raf, which have a binding domain of Ras in the N-terminal regulatory region [12, 13]. Raf molecule belongs to protein serine/threonine protein kinase, which catalyzes the phosphorylation and activation of MEK1/2 and further catalyzes the phosphorylation and activation of ERK [14]. The first Raf subtype in Raf kinases, C-Raf, is also known as Raf-1 [12]. Recently, one study showed that the activation of C-Raf/MEK/ERK signaling pathway promoted cell proliferation in cervical cancer [15]. Another study has also found that activation of C-Raf/MEK/ERK pathway was responsible for the proliferation and migration of cervical cancer cells [16]. These findings suggest that C-Raf/MEK/ERK signaling pathway plays a pivotal role in cell proliferation and cancer progression. In our current study, the expression levels of TM7SF2 and its related carcinogenic effects and underlying biological mechanisms in cervical cancer were uncovered. The results of our study suggest that TM7SF2/C-Raf/ERK1/2 axis exerts an essential effect on the progression of cervical cancer and targeting TM7SF2 may be a promising method to treat cervical cancer.

## Results

### TM7SF2 is highly expressed in cervical cancer cells and tissues

**Fig. 1:**
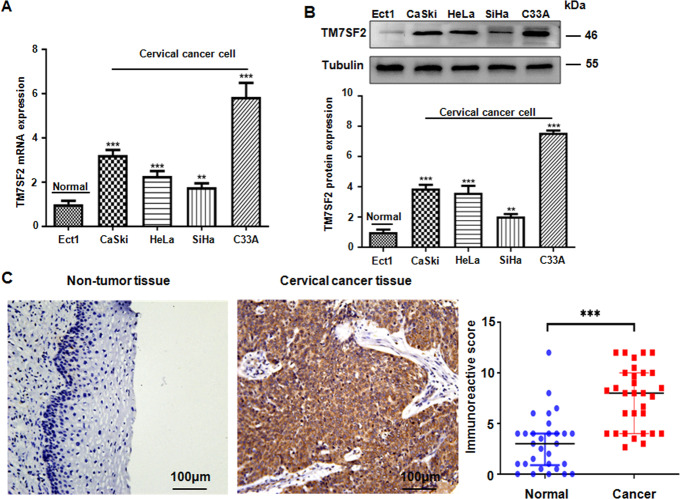
The expression of TM7SF2 is upregulated in cervical cancer. **A** The expression level of TM7SF2 mRNA was measured by real-time RT-PCR in human cervical cancer cells compared with normal cervical epithelial cells. **p < 0.01, ***p < 0.001 compared to Ect1 cells. **B** Top panel: the expression level of TM7SF2 protein was measured by western blotting in human cervical cancer cells compared with normal cervical epithelial cells. Bottom panel: quantitative results were illustrated. **p < 0.01, ***p < 0.001 compared to Ect1 cells. **C** Left panel: the expression level of TM7SF2 protein was measured by IHC in human cervical cancer tissues and adjacent non-tumor tissues. Right panel: IHC scores were used to analyze the experimental results. The data were displayed as median with interquartile range. ***p < 0.001 compared to normal tissues.

The expression of TM7SF2 at the transcriptional and translational levels was detected in normal cervical epithelial cell line Ect1/E6E7 and cervical cancer cell lines, including C33A, SiHa, CaSki, and HeLa. As shown in Fig. 1A, B, the expression of TM7SF2 at mRNA and protein levels was notably elevated in cervical cancer cells compared with Ect1/E6E7 cells by use of RT-PCR and western blotting. In line with the expression of TM7SF2 in cervical cancer cells, higher expression of TM7SF2 protein in cervical cancer tissues was observed in Fig. 1C. The color of brown staining was regarded as positive expression. The immunohistochemistry staining results indicated that the expression of TM7SF2 was increased in cervical cancer tissues in comparison with the corresponding normal tissues adjacent to the cancer. In conclusion, TM7SF2 expression was evidently increased in human cervical cancer tissues and cells.

### TM7SF2 promotes the proliferation of human cervical cancer cells

**Fig. 2:**
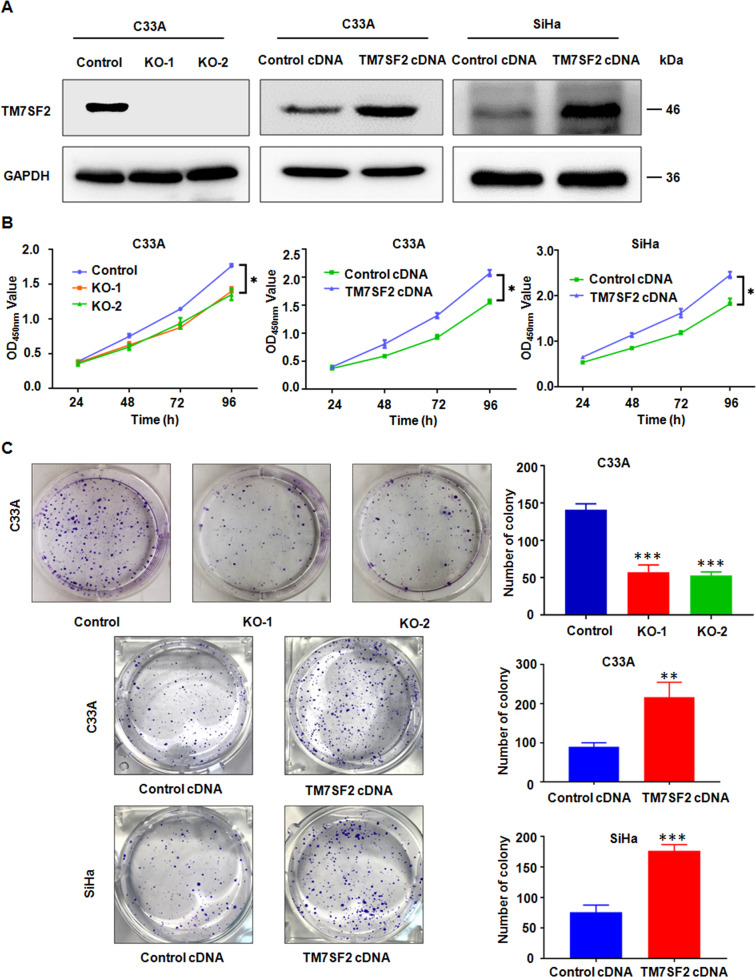
TM7SF2 promotes cell proliferation and colony formation in cervical cancer cells. **A** The efficacy of TM7SF2 knockout or overexpression in C33A and SiHa cells were measured by western blotting. **B** The effect of TM7SF2 knockout or overexpression on cell proliferation ability was measured by CCK-8 assays in cervical cancer cells. *p < 0.05 compared to control. **C** Left panel: the effect of TM7SF2 knockout or overexpression on colony formation in cervical cancer cells. Right panel: quantitative analysis for left panel. **p < 0.01, ***p < 0.001 compared to control.

Due to the relatively high expression of TM7SF2 in C33A cell line, we established a stable TM7SF2-knockout C33A cell line to figure out the biological functions of TM7SF2 in cervical cancer. In addition, TM7SF2 was ectopically overexpressed in SiHa cells with relatively low expression of TM7SF2. Meanwhile, the overexpression of C33A cells was also established in order to further explore the effects of TM7SF2 on cervical cancer cells. The efficacy of TM7SF2 knockout and overexpression in these constructed cell lines was verified (Fig. 2A). Consequently, the cell proliferation by CCK-8 assay and colony formation assay were explored. As shown in Fig. 2B, C, compared with the vector control group, cell viability and colony formation ability were strongly suppressed in C33A cells after TM7SF2 knockout. In contrast, TM7SF2 overexpression promoted cell proliferation and colony formation in C33A and SiHa cells (Fig. 2B, C). These data suggest that functional experiments have uncovered that TM7SF2 could effectively facilitate cell growth and may be a therapeutic target for cervical cancer.

### TM7SF2 contributes to promoting cell migration and invasion

**Fig. 3:**
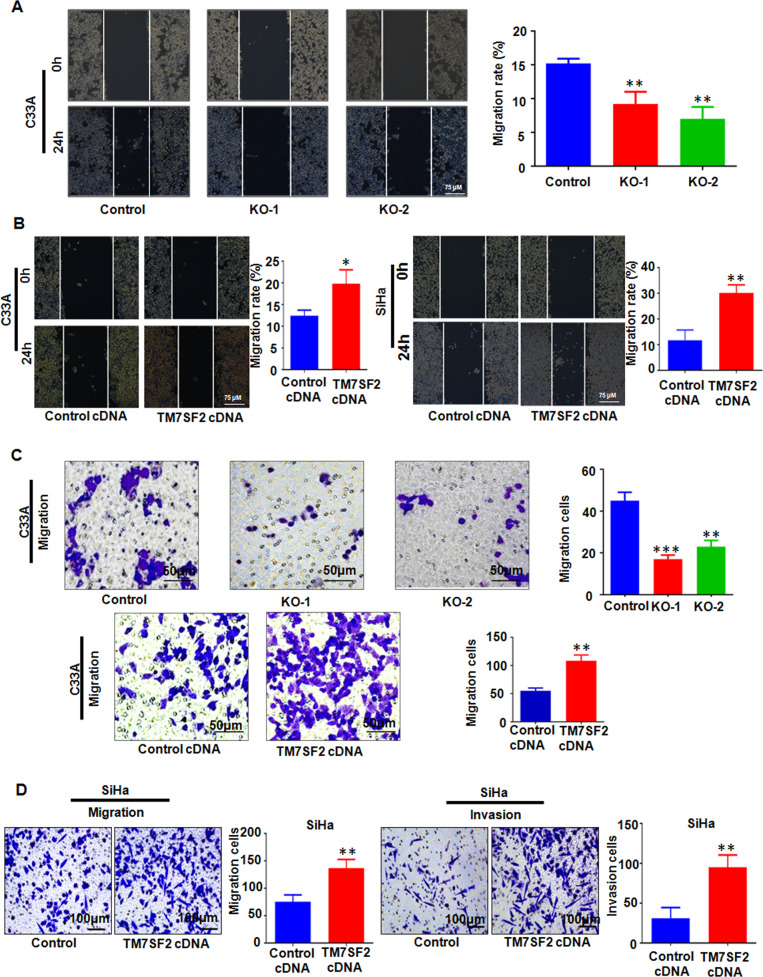
TM7SF2 promotes migration and invasion of cervical cancer cells. **A** Left panel: the effect of TM7SF knockout on wound healing in C33A cells. Right panel: quantitative analysis for left panel. **p < 0.01 compared to control. **B** Left panel: the effect of TM7SF overexpression on wound healing in C33A and SiHa cells. Right panel: quantitative analysis for left panel. *p < 0.05, **p < 0.01 compared to control. **C** Left panel: the effect of TM7SF knockout or overexpression on migration by Transwell assay in C33A. Right panel: quantitative analysis for left panel. **p < 0.01, ***p < 0.001 compared to control. **D** Left panel: the effect of TM7SF overexpression on migration and invasion by Transwell assay in SiHa cells. Right panel: quantitative analysis for left panel. **p < 0.01 compared to control.

We further explored whether TM7SF2 contributed to motility of cervical cancer cells. Wound healing assay data showed that the number of migration cells was decreased after TM7SF2 knockout, while overexpression of TM7SF2 enhanced wound area closure (Fig. 3A, B). Next, we examined the role of TM7SF2 in migration and invasion of C33A and SiHa cell. Our results found that overexpression of TM7SF2 enhanced migration and invasion ability of SiHa cells and promoted the migration ability of C33A cells by wound healing assay and Transwell assay (Fig. 3C, D). Above all, these findings revealed that TM7SF2 overexpression obviously promoted migration and invasion of cervical cancer cells.

### Inhibition of TM7SF2-induced G0/G1 phase arrests and apoptosis

**Fig. 4:**
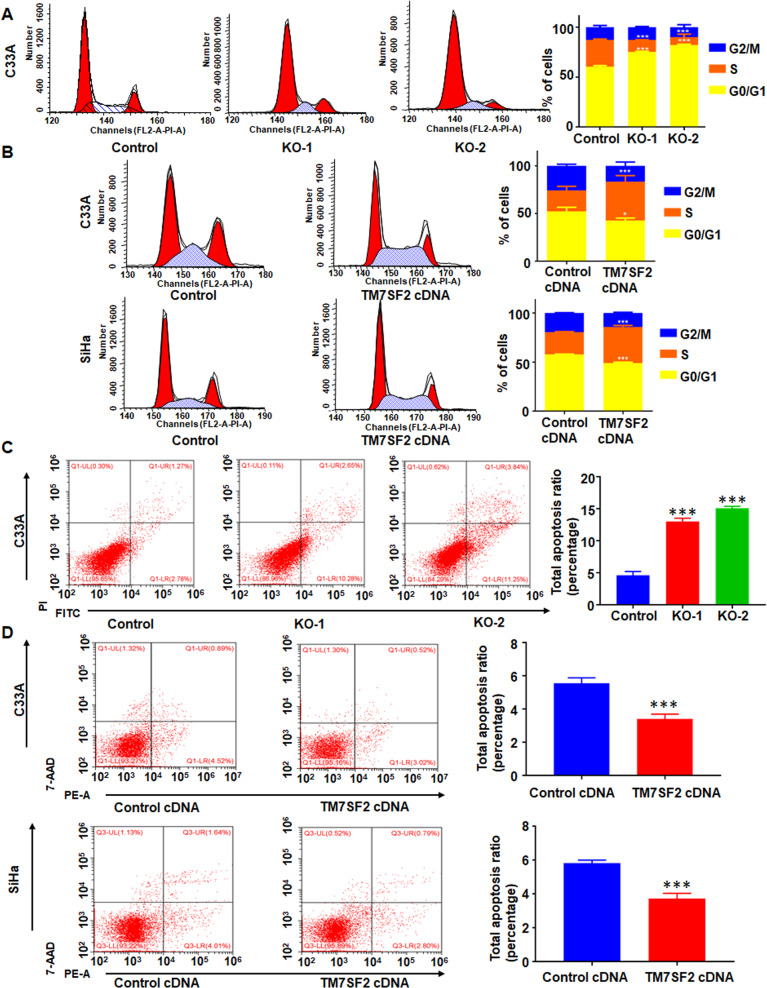
TM7SF2 modulates cell cycle distribution and apoptosis in cervical cancer cells. **A** Left panel: the effect of TM7SF knockout on cell cycle distribution in C33A cells. Right panel: quantitative analysis for left panel. **B** Left panel: the effect of TM7SF overexpression on cell cycle distribution in C33A and SiHa cells. Right panel: quantitative analysis for left panel. **C** Left panel: the effect of TM7SF knockout on cell apoptosis in C33A cells. Right panel: quantitative analysis for left panel. ***p < 0.001 compared to control. **D** Left panel: the effect of TM7SF overexpression on cell apoptosis in C33A and SiHa cells. Right panel: quantitative analysis for left panel. ***p < 0.001 compared to control.

**Fig. 5:**
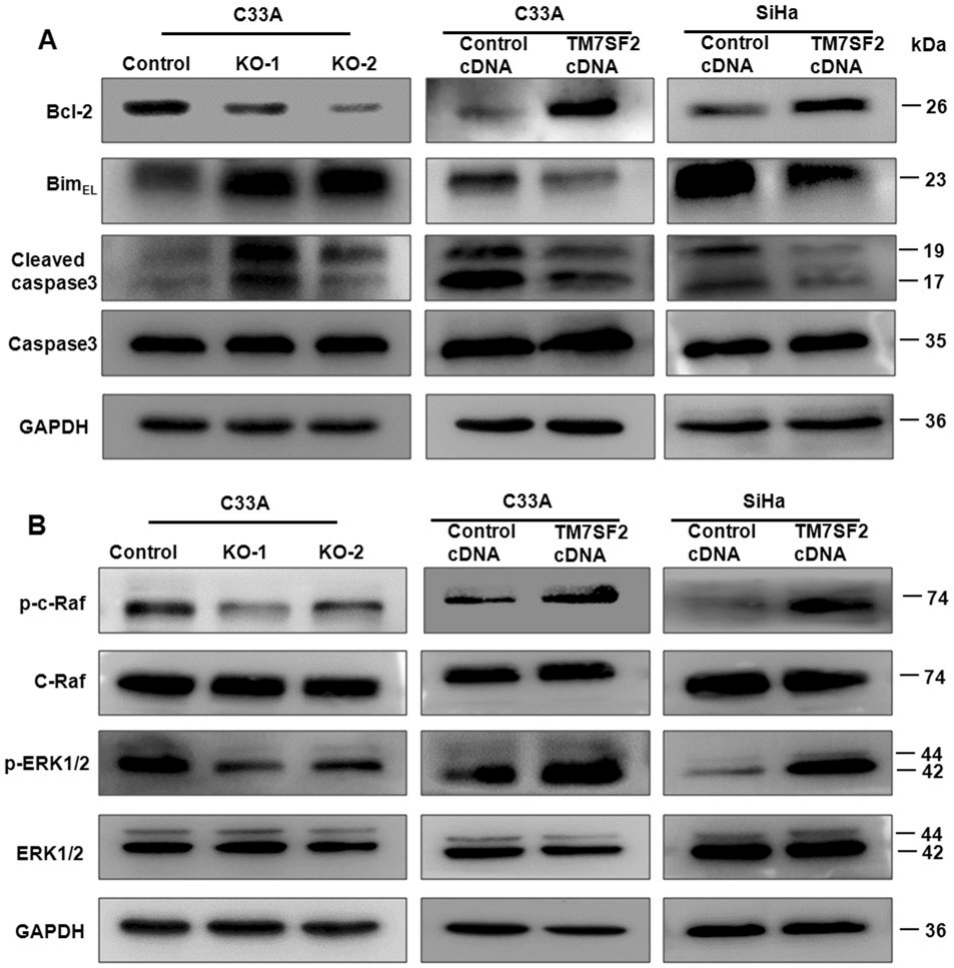
TM7SF2 modulates the expression of apoptosis related proteins and C-Raf/ERK pathway. **A** Western blotting was utilized for measuring the expression of apoptosis related proteins in cells after TM7SF2 knockout or overexpression. **B** Western blotting was conducted to detect the effect of TM7SF2 knockout or overexpression on C-Raf/ERK signaling pathway.

The cell cycle distribution was evaluated by flow cytometry. Compared with the control group, TM7SF2-knockout C33A cells displayed prolonged G0/G1 phase and shorten S phase. These results suggest that knockout of TM7SF2 leads to cell cycle arrest at the G0/G1 phase (Fig. 4A). On the other hand, TM7SF2 overexpression in C33A and SiHa cells decreased the ratio of G0/G1 phase and increased S phase (Fig. 4B). After that, we investigated the rate of apoptosis using flow cytometry in cervical cancer cells after TM7SF2 modulation. As demonstrated in Fig. 4C, the number of apoptotic death cells was increased after knockout of TM7SF2 in C33A cells. In contrast, overexpression of TM7SF2 decreased the apoptosis of C33A and SiHa cells (Fig. 4D). Subsequently, western blotting assays unveiled that the upregulation of apoptosis markers, such as Cleaved-caspase 3 and Bim_EL_, as well as downregulation of Bcl-2 in TM7SF2-knockout C33A cells were observed. However, in TM7SF2-overexpressing C33A and SiHa cells, the results displayed downregulation of Cleaved-caspase 3 and Bim_EL_, as well as upregulation of Bcl-2 (Fig. 5A). These data strongly implied that TM7SF2 may be a tumor promoter in cervical cancer.

### TM7SF2 promotes cell proliferation and inhibits apoptosis via C-Raf/ERK signaling pathway

**Fig. 6:**
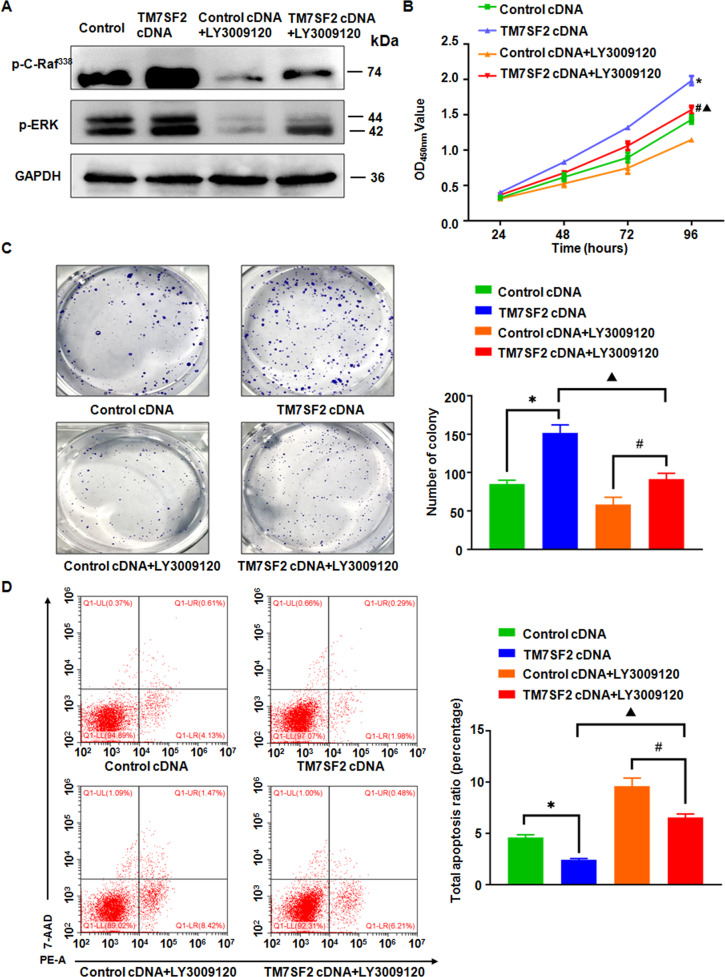
TM7SF2 modulates cell proliferation and apoptosis via C-Raf/ERK signaling pathway in cervical cancer cells. **A** The inhibitory effect of C-Raf/ERK pathway after treatment with Raf inhibitor LY3009120 in C33A cells. **B**, **C** CCK-8 assay and colony formation assay showed that LY3009120 reversed TM7SF2-induced promotion of proliferation in C33A cells. **D** The flow cytometry assay showed that LY3009120 treatment rescued the suppression of apoptosis by TM7SF2 overexpression in C33A cells. *p < 0.05 compared to control cDNA, #*p* < 0.05 compared to control cDNA+ LY3009120, Δ*p* < 0.05 compared to TM7SF2 cDNA.

To figure out the underlying mechanism of TM7SF2 in cervical tumorigenesis, we further investigated that whether C-Raf/ERK1/2 signaling pathway is involved in TM7SF2-induced cervical cancer. The regulation of C-Raf/ERK1/2 signaling pathway by TM7SF2 was confirmed in cervical cancer cells. In TM7SF2-knockout C33A cells, p-C-Raf (S338) and p-ERK1/2 (T202/Y204) expressions were reduced (Fig. 5B). In TM7SF2-overexpressing C33A and SiHa cells, p-C-Raf (S338) and p-ERK1/2 (T202/Y204) expressions were increased (Fig. 5B). Multiple studies have demonstrated that the C-Raf/ERK signaling pathway plays a pivotal role in cells proliferation and apoptosis [17, 18]. Next, the 1 μM Raf inhibitor LY3009120 was utilized to explore the role of C-Raf/ERK signaling pathway in TM7SF2-mediated cervical oncogenesis. Western blotting results showed that LY3009120 inhibited the activity of C-Raf/ERK signaling pathway in TM7SF2-overexpressing C33A cells (Fig. 6A). After treating with LY3009120 in TM7SF2-overexpressing C33A cells, the capabilities of cell viability and colony formation were reversed partly (Fig. 6B, C). Besides, the apoptosis of TM7SF2-overexpressing C33A cells was induced after treatment with LY3009120 (Fig. 6D). These data indicated that C-Raf/ERK signaling pathway exerted an essential influence in cervical cancer development.

### TM7SF2 overexpression contributes to xenograft tumor growth in vivo

**Fig. 7:**
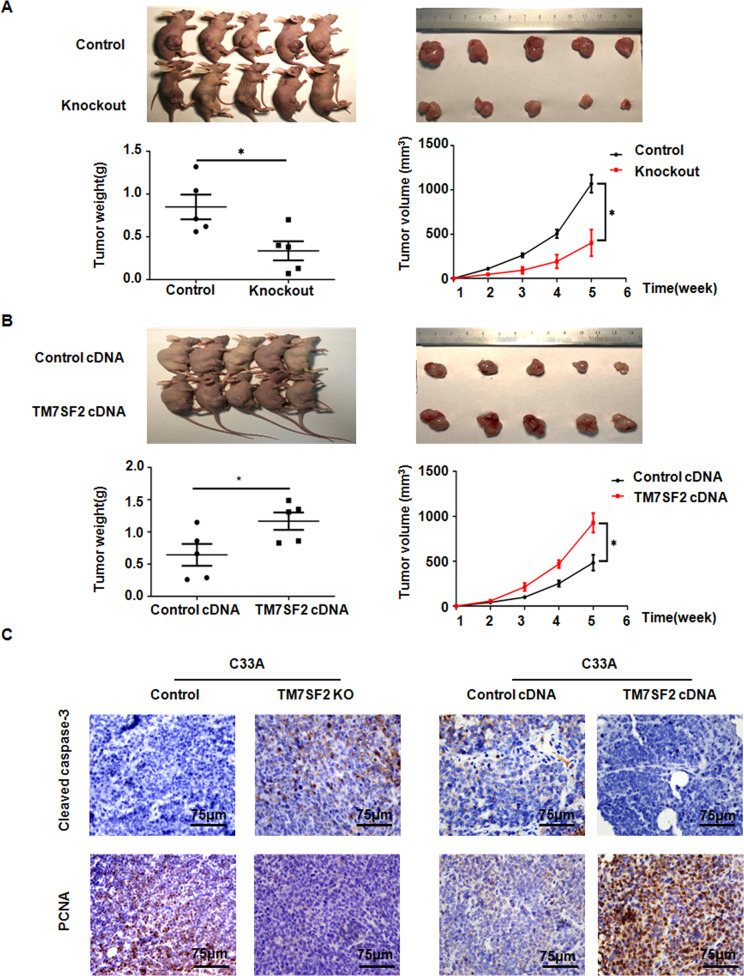
TM7SF2 promotes the subcutaneous tumor growth in vivo. **A**, **B** Upper: the pictures of nude mice and xenografts were collected and photographed at the 35th day after subcutaneous injection of TM7SF2-overexpressing or TM7SF2-knockout C33A cells. Bottom: the weight of each tumor was measured and the tumor volume was calculated according to the formula (L × W^2^)/2. The data were showed as mean ± SEM. **C** The representative images for immunohistochemical staining of Cleaved caspase-3 and PCNA in C33A subcutaneous xenografts with TM7SF2-overexpressing or TM7SF2-knockout groups. *p < 0.05 compared to control.

To further verify the tumor promotive role of TM7SF2 in cervical cancer, TM7SF2-KO C33A cells and TM7SF2-OE C33A cells were injected subcutaneously into female nude mice. It was found that the sizes and weights of xenografts were obviously decreased in the TM7SF2-KO group compared with control group (Fig. 7A). On the other hand, the tumor sizes and weights of the TM7SF2-OE group xenografts were significantly greater than that in the vector control group (Fig. 7B). Furthermore, we found that the proliferation marker PCNA was strongly upregulated in tumor sections from TM7SF2-OE nude mice group but PCNA expression was decreased in TM7SF2-KO nude mice group (Fig. 7C). Immunohistochemical (IHC) staining also showed that tumor sections from nude mice that injected subcutaneously with TM7SF2-KO C33A cells expressed more apoptotic marker Cleaved caspase-3, while TM7SF2-OE C33A cells showed lower expression of Cleaved caspase-3 (Fig. 7C). In summary, TM7SF2 promoted tumor growth and inhibited apoptosis in vivo, contributing to the progression of cervical cancer.

## Discussion

Cervical cancer is the fourth common malignant tumor in women, and the increasing risk of cervical cancer among young women in some areas has emerged [19, 20]. It is an urgent need to further explore its underlying molecular mechanism and develop promising molecular targeted drugs [1]. The cancer occurrence is a complex, multi-factor, and multi-step regulatory process and the dysfunction of oncogene or antioncogene in cancer cells plays a vital role in the proliferation, invasion, and migration of cancer cells. In general, inhibiting tumor growth and promoting apoptosis might be a promising strategy for cancer therapy. In our study, we found that the expression of TM7SF2 mRNA and protein was upregulated in cervical cancer cells compared with normal cervical epithelial cells. Consistently, TM7SF2 protein was increased in cervical cancer tissues compared with its corresponding adjacent normal cervical tissues.

One study found that the downregulation of TM7SF2 would inhibit liver cell proliferation and restrained cells from G1 phase to S phase. They also uncovered that the absence of TM7SF2 impaired the regeneration of liver in *TM7SF2* KO mice, which may be due to an increased expression of p53 and p21 [10]. In order to further figure out the function of TM7SF2 in cervical cancer cells, TM7SF2-knockout and TM7SF2-overexpressing cells were constructed in C33A and SiHa cells. In TM7SF2-knockout C33A cells, the cell proliferation and migration were suppressed but cell apoptosis was increased, whereas TM7SF2-overexpressing C33A and SiHa cells exhibited the opposite results. Moreover, TM7SF2 regulated the distribution of cell cycle. The dysfunction of cell apoptosis is essential in the development and progression of human malignancies, including the activation of anti-apoptotic protein and the inhibition of apoptotic protein [21]. Previous study has revealed that Bcl-2 family plays a key role in apoptotic pathway. Bcl-2 is an inhibitor of apoptosis while Bim promotes the cell apoptosis [22]. Collectively, cleaved caspase 3 induced cell apoptotic responses [23]. Our results revealed that the expression of apoptosis related protein Bim_EL_, Cleaved caspase 3 was increased, while Bcl-2 was decreased in TM7SF2-knockout cells. On the contrary, the apoptosis related protein was down-regulated, while anti-apoptosis related protein was upregulated in TM7SF2-overexpressing cells.

C-Raf, a serine-threonine kinase, is an important molecule in the regulation of cell proliferation and survival [24]. Similar to C-Raf, the activation of ERK1/2 also promoted cell growth, migration, invasion, and inhibited cell apoptosis in cervical cancer [25, 26]. Furthermore, a wealth of studies demonstrated that C-Raf/ERK1/2 signaling pathway contributed to facilitating cancer progression [17, 18]. One study demonstrated activation of C-Raf/ERK signaling pathway via phosphorylating C-Raf on serine 338 and ERK1/2 on threonine 202 and tyrosine 204 in esophageal squamous cell cancer [27]. In the present study, we found that TM7SF2-knockout inhibited C-Raf/ERK1/2 signaling pathway but TM7SF2 overexpression activated this signaling pathway. Next, in order to further illustrate the biological mechanism of TM7SF2 via C-Raf-ERK pathway in the progression of cervical cancer, we used an inhibitor to suppress the C-Raf/ERK1/2 pathway. LY3009120, a pan inhibitor of Raf, induced cell apoptosis and cell cycle arrest at G0/G1 phase while inhibited cancer cell proliferation [28, 29]. Our results revealed that after treatment of TM7SF2-overexpressing C33A cells with LY3009120, the cell proliferation was inhibited and cell apoptosis was trigged. LY3009120 abrogated the promotive effect of TM7SF2 on cervical cancer cells. These data uncovered that TM7SF2 promoted cell proliferation and reduced cell apoptosis by C-Raf/ERK1/2 pathway.

Using in vivo animal experiment, we also found that TM7SF2 overexpression promoted the growth of subcutaneous xenografts. PCNA was a key regulator in multiple processes, such as DNA synthesis, DNA damage avoidance, and DNA repair [30]. Cleaved caspase 3 was an essential modulator of the cell apoptosis [23]. Importantly, our study found that TM7SF2 overexpression increased the expression of PCNA protein and decreased Cleaved caspase-3 protein in nude mice xenografts. Hence, TM7SF2 may be an oncogene in cervical cancer by in vivo and vitro experiments.

## Conclusion

**Fig. 8:**
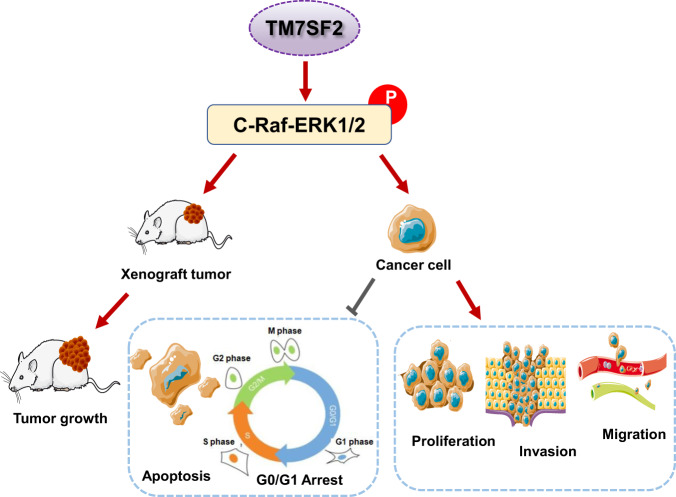
A graphical diagram illustrates how TM7SF2 promotes the tumorigenesis of cervical cancer. TM7SF2 involves in promotion of cell proliferation, migration, invasion, tumor growth as well as inhibition of cell apoptosis and cell cycle progression. TM7SF2 triggers cervical tumorigenesis via the activation of the C-Raf-ERK pathway, and then promotes cell growth and inhibits apoptosis in cervical cancer.

In summary, we reported that the tumor promotive functions of TM7SF2 in cervical cancer. TM7SF2 is an essential molecule involving in the activation of C-Raf-ERK pathway and then promotes cell growth and inhibited apoptosis in cervical cancer (Fig. 8), indicating that inhibition of TM7SF2 may be a therapeutic strategy in the future for cervical cancer patients.

## Materials and methods

### Tissue samples

Tissue microarray was purchased from Shanghai Outdo Biotech Company (Shanghai, China) with the approval of the Institutional Review Board, which included 31 cervical cancer cases and 30 corresponding adjacent normal cervical tissues in order to evaluate the expression of TM7SF2 in cervical cancer tissues.

### Immunohistochemical staining

The paraffin blocks were cut into 4 μm slices and dried at 60 °C for 30 min. Then antigen retrieval was performed by heating the sections using microwave in 10 mM citrate buffer (pH 6.0) for 10 min followed by cooling at room temperature for 1 h. After that, each section was incubated with primary antibodies for anti-TM7SF2 antibody (1:400, Biorbyt, orb4574), anti-PCNA antibody (1:100, Abcam, ab29), and anti-Cleaved caspase3 antibody (1:100, CST, 9664 S) overnight at 4 °C. Next, the slides were washed twice with PBS and then treated with HRP-conjugated secondary antibody at room temperature for 30 min. Afterwards, the sections were stained using 3,3-diaminobenzidine as a brown chromogen for 2 min. Finally, the cell nuclei were counterstained by use of hematoxylin for 20 s. Staining intensity score was classified into 0 (no staining), 1 (weak staining), 2 (moderate staining), and 3 (strong staining). Staining frequency score of each sample was graded as follows: 0, <5%; 1, 6–25%; 2, 26–50%; 3, 51–75%; 4, >75%. The protein expression was scored semi-quantitatively by multiplying the staining intensity and staining frequency, as well as each image result was determined by two pathologists using light microscopy.

### Cell culture

The cervical cancer cell lines C33A, SiHa, CaSki, and HeLa, as well as the normal cervical epithelia cells Ect1/E6E7 were obtained from ATCC (American Type Culture Collection). C33A, SiHa, HeLa, and Ect1/ E6E7 cells were cultured with Dulbecco’s modified Eagle’s medium (DMEM; Gibco, USA) and CaSki cells were cultured in Roswell Park Memorial Institute (RPMI)−1640 with the addition of 10% Fetal Bovine Serum (FBS) and 1% antibiotics (streptomycin/penicillin; Gibco, USA) under 5% CO_2_ at 37 °C in a humidified incubator.

### RNA extraction and qRT-PCR

Cells were washed with cold PBS three times and the total RNA extracted immediately with 1 ml TRIzol Reagent (Thermo Fisher Scientific, USA) by following the manufacturer’s instructions. In all, 1 μg RNA was used as a template to reversely transcribe into first-strand cDNA. After that, the qRT-PCR was performed with the SYBR Green kit (TaKaRa, Japan) based on the standard protocols. The primers were as follows: TM7SF2 (Forward 5′−3′: CAG CAT GAA GCC AAA CCC; Reverse 5′−3′: TCT GTG AAC TGC GAC CCG); GAPDH (Forward 5′−3′: AAG AAG GTG GTG AAG CAG G; Reverse 5′−3′: GTC AAA GGT GGA GGA GTG G). The mRNA expression levels of target genes were normalized to GAPDH mRNA expression levels. The gene expression was analyzed with 2^−∆∆CT^ method.

### Western blotting analysis

Cells were lysed with cell lysis buffer containing phenylmethanesulfonylfluoride (PMSF). Total amount of protein lysate (40 µg) was separated on SDS/PAGE, and then proteins were electrophoretically transferred for 90 min onto a polyvinylidene difluoride (PVDF) membrane. Membranes were blocked with 5% nonfat milk for 2 h and incubated with the primary antibodies overnight at 4 °C. The primary antibodies were as follows: anti-TM7SF2 rabbit polyclonal antibody (1:1000, Biorbyt, orb4574), anti-Bcl2 rabbit monoclonal antibody (1:1000, CST, 4223S), anti-Bim rabbit monoclonal antibody (1:1000, CST, 2933S), anti-Caspase3 rabbit antibody (1:1000, CST, 9662S), anti-Cleaved caspase3 rabbit monoclonal antibody (1:1000, CST, 9664S), anti-phospho-C-Raf rabbit monoclonal antibody (1:1000, CST, 9427T), anti-C-Raf rabbit antibody (1:1000, CST, 9422T), anti-phospho-ERK1/2 rabbit monoclonal antibody (1:1000, CST, 4370S), anti-ERK1/2 rabbit monoclonal antibody (1:1000, CST, 4695S), and anti-GAPDH mouse monoclonal antibody (1:1000, Proteintech, 60004-1-Ig). Then the PVDF membranes were washed three times with TBST and incubated with secondary antibody for 1.5 h at room temperature and they were visualized using the Enhanced chemiluminescent (ECL) detection reagents. ImageJ software was used for band quantification analysis.

### CCK8 cell viability assay

Cell viability was measured by use of the Cell Counting Kit-8 kit (Beyotime, Shanghai, China). C33A cells (1 × 10^4^ cells per well) and SiHa (2 × 10^3^ cells per well) were seeded on 96-well plates in DMEM supplemented with 10% FBS. After the cells were incubated for 1, 2, 3, and 4 days, respectively, 10 μL CCK-8 reagent was added to each well and incubated at 37 °C for 3 h. Finally, the OD values were assessed at a wavelength of 450 nm for each well using a microplate reader.

### Colony formation assay

The cervical cancer cells were seeded into six-well plates (800 cells/well) and cultured the cells with DMEM containing 10% FBS for 10 days in 37 °C at 5% CO_2_ incubator. Next, the culture media was discarded and the cells were washed with PBS three times. Afterwards, we fixed the cells with 4% paraformaldehyde for 30 min. Finally, the cell colony was stained by use of 0.1% crystal violet for 10 min. The number of cell colony was calculated.

### Apoptosis and cell cycle assay

For the cell apoptosis assay, we used the FITC-labeled Annexin V (Annexin V-FITC) apoptosis detection kit (BD Biosciences) to examine apoptotic death cells on the basis of the manufacture’s protocol. The cells were digested with EDTA-free trypsin and collected in stream tubes. For cells with GFP fluorescence, cells were harvested and stained with 5 μl Annexin-PE and 10 μl 7-AAD following the manufacturer’s instructions. For cells without GFP fluorescence, cell suspension was added 5 μl FITC Annexin V and 5 μl PI. In addition, for the cell cycle assay, it was performed using the Cell Cycle Kit. Cells were harvested, washed once with PBS, and fixed the cells with 3 ml of 75% precooling ethanol at −20 °C overnight. The next day, the cells were washed with PBS two times and incubated with 500 μl RNase and PI reagents for half an hour at room temperature in the dark. Cell cycle was analyzed by the Modfit LT software.

### Cell transfection

For the overexpression of TM7SF2 in C33A and SiHa cells, the TM7SF2 cDNA sequence was cloned into pCDH-GFP + Puro vector, which was obtained from Changsha Youbio Biosciences Inc. The lentivirus particles were produced by transfecting the corresponding vectors with helper plasmids pMD2.G and psPAX2 in HEK293T cells. Next, the virus infected target cells and stable TM7SF2-overexpressing cells were generated after selection with 2 μg/ml puromycin.

### Generation of TM7SF2 knockout clones by CRISPR/Cas9 technique

TM7SF2 knockout in C33A cells was produced by CRISPR-Cas9 technique. Cells were transfected with 2 µg of each sgRNA. After 48 h post-transfection, cells were selected by 2 µg/mL puromycin. Then, the cells were diluted into single cell and seeded into 96-well plate, and confirmed the efficacy of knockout by western blotting analysis. TM7SF2-knockout C33A cells were transfected with pSpCas9(BB)−2A-Puro(pX459)-TM7SF2 sgRNA. The TM7SF2 oligonucleotide sequences were showed in the following: TM7SF2-sgRNA1-F: CAC CGC AGG CGG CGC TCT ACC TAC; TM7SF2-sgRNA1-R: AAA CGT AGG TAG AGC GCC GCC TGC; TM7SF2-sgRNA2-F: CAC CGC CTG CTC CTG GCG GCC CGT T; TM7SF2-sgRNA2-R: AAA CAA CGG GCC GCC AGG AGC AGG C.

### Transwell assay

The Transwell chambers (Millipore, USA) were utilized to evaluate the migratory and invasive capabilities of the cells. The cells were planted in serum-free DMEM to a density of 1 × 10^5^~1 × 10^6^ cells/ml with 100 μl in Transwell plates (8 µm pore size) in a 24-well plate. Nevertheless, 500 μl DMEM containing 10% FBS was filled at the bottom of a 24-well plate, contributing to the upper cells across the Transwell membrane into the lower chamber. For invasion assay, the upper compartments were pre-coated with Matrigel (BD Biosciences, CA). After 24 h incubation, the culture medium in the upper chamber were discarded and the Transwell chambers were washed with PBS two times, then chambers were fixed with 4% paraformaldehyde for 30 min before staining with 0.1% crystal violet. The migrated or invaded cells were counted under an inverted routine microscope (Nikon Instruments Inc) in five random fields.

### Wound healing assay

The migration ability was detected by the wound healing assay. The wound was scratched by pipette tip when cell confluence reached to >90%. At 0 h and 24 h, the wound area was photographed and analyzed with image J software (NIH, USA).

### Antitumor study in vivo

Five-week-old female nude mice (BALA/c-nu, Beijing Weitonglihua Sciences Co. Inc., China) were purchased and placed in a specific pathogen-free (SPF) room and adapted for ~1 week. After that, 5 × 10^6^ cells with gene alternation (including TM7SF2-overexpressing C33A cells, TM7SF2-knockout C33A cells, and their respective control groups) were injected subcutaneously with 100 μl PBS into the right flanks of the nude mice (5 mice per group). Tumor growth of each mice was calculated by caliper twice a week for 35 days, and the volume of each tumor was measured by the formula (volume = length × (width^2^)/2). After 35 days of injection, all the nude mice were euthanized, and then the xenograft tumors were removed and weighed. Finally, the xenografts were fixed with 4% formaldehyde, dehydrated, paraffin-embedded, and cut into 4 μm of slices. All the animal experiments were approved by the Institutional Animal Care and Use Committee of Wenzhou Medical University.

### Statistical analysis

Normally distributed results are displayed as the mean ± standard deviation (SD) unless otherwise illustrated in the figure legends. When the data conforms to the normally distribution, the Student’s *t* test was used to compare between two groups, and ANOVA is used for the comparison in multiple groups. Levene’s Test was used to test whether or not the variance among two or more groups is equal. For non-normally distributed data, the Mann–Whitney testing was used to compare the statistical significance of variations. All the statistical data were analyzed by utilizing GraphPad Prism 8.0. Differences of *P < 0.05 *, **P < 0.01 or ***P < 0.001 were represented statistical significance.

## Supplementary information

Author contribution form

## Data Availability

The datasets used and analyzed during the current study are available from the corresponding author on reasonable request.
