# Supplementary material for: TM7SF2 regulates cell proliferation and apoptosis by activation of C-Raf/ERK pathway in cervical cancer
Source: Cell Death Discov. 2021 Oct 19;7:299. doi: 10.1038/s41420-021-00689-5 (PMC8526692; doi:10.1038/s41420-021-00689-5)
Supplement: Supplementary file 1 — Author contribution form [file 41420_2021_689_MOESM1_ESM.pdf]

DECLARATION OF CONTRIBUTIONS TO ARTICLE

ADMC

Manuscript Number: CDDISCOVERY-21-2386

Journal Name: *Cell Death Discovery* (the 'Journal')

Proposed Title of the Contribution:

TM7SF2 regulates cell proliferation and apoptosis by activation of C-Raf/ERK pathway in cervical cancer (the 'Contribution)

Author(s):

Yichi Xu, Xin Chen, Shuya Pan, Zhi-wei Wang, Xueqiong Zhu (the 'Authors')

For all *CDDis*covery articles, each person named as an author in the published version must be able to show he or she has contributed substantially to the article.

Authorship credit should be based on 1) substantial contributions to conception and design, acquisition of data, or analysis and interpretation of data; 2) drafting the article or revising it critically for important intellectual content; and 3) final approval of the version to be published. Authors should meet conditions 1, 2 and 3.

Any person who cannot be shown to have made a substantial contribution to the article cannot be listed as an author in the final version. The name of any person who is deemed to have made a minor contribution can, however, appear in the Acknowledgments section of the article.

Please complete the table below to indicate the contributions of all named authors to the manuscript.

Author Full Name:	Specification of Contribution to the Manuscript:
Yichi Xu	acquisition of data, analysis and interpretation of data, drafting the article, final approval of the version to be published
Xin Chen	acquisition of data, analysis and interpretation of data, final approval of the version to be published
Shuya Pan	analysis and interpretation of data, final approval of the version to be published
Zhiwei Wang	substantial contributions to conception and design, final approval of the version to be published
Xueqiong Zhu	drafting the article, substantial contributions to conception and design, final approval of the version to be published

Please complete the table below to indicate the contributions of all named authors to the figures.

Figure 1:

Yichi Xu

Figure 2:

Yichi Xu, Xin Chen

Figure 3:

Yichi Xu, Xin Chen

Figure 4:

Yichi Xu

Figure 5:

Yichi Xu

Figure 6:

Yichi Xu, Xin Chen for Fig. 6, 7 and 8.

Signed for and on behalf of the Author(s):	Print Name:	Date:
Wang	Zhiwei Wang	Sept 6, 2021
